# Bilateral Tubo‐Ovarian Abscess as an Unusual Trigger of Diabetic Ketoacidosis in a Woman With Type 2 Diabetes: A Rare Case Report

**DOI:** 10.1155/crog/4323528

**Published:** 2026-07-01

**Authors:** Hala O. Abdallah, Bara J. Abufara, Hobab J. Odeh, Ansam Nafah, Reem Shihab, Mua′weah Shroof

**Affiliations:** ^1^ Department of Medicine, Faculty of Medicine and Health Sciences, An-Najah National University, Nablus, State of Palestine, najah.edu; ^2^ Obstetrics and Gynecology Department, Istishari Arab Hospital, Ramallah, State of Palestine

**Keywords:** diabetic ketoacidosis, tubo-ovarian abscess, Type 2 diabetes mellitus

## Abstract

**Background:**

Tubo‐ovarian abscess is a serious complication of pelvic inflammatory disease that often requires surgical management. Diabetic ketoacidosis is a life‐threatening metabolic emergency most seen in Type 1 diabetes but can rarely occur in Type 2 diabetes. The simultaneous occurrence of TOA and DKA is extremely rare, highlighting a clinically important association.

**Case Presentation:**

We report a 37‐year‐old woman with poorly controlled Type 2 diabetes mellitus who presented with severe abdominal pain, fever, and metabolic decompensation diagnosed as DKA. On workup, CT scan revealed bilateral tubo‐ovarian abscesses. She was admitted to the intensive care unit and managed appropriately. Persistent fever with elevated CRP prompted laparoscopic drainage of both abscesses, resulting in clinical improvement.

**Conclusion:**

Bilateral tubo‐ovarian abscess can precipitate diabetic ketoacidosis in women with Type 2 diabetes mellitus. Recognizing gynecologic sepsis as a potential precipitant is crucial for timely management.

## 1. Introduction

Tubo‐ovarian abscess (TOA) is a serious adnexal infection characterized by the accumulation of pus within the fallopian tube and ovary. It is a severe complication of untreated pelvic inflammatory disease (PID), occurring in approximately 15%–35% of PID cases [1] and affecting about 2.3% of women overall, with nearly 46% being bilateral [2]. TOA typically occurs in sexually active women of reproductive age, particularly those with risk factors such as multiple sexual partners, intrauterine device use, in vitro fertilization, or a history of sexually transmitted infections (STIs). Diagnosis relies on clinical features—fever, pelvic pain, and abnormal discharge—supported by raised inflammatory markers, for example, WBC count, ESR, CRP, and imaging findings. Management ranges from broad‐spectrum antibiotics to drainage or surgical intervention, depending on abscess size and clinical severity [3].

## 2. Case Presentation

A 37‐year‐old woman (G4P3A1) presented with a 1‐week history of progressively worsening diffuse abdominal pain associated with nausea, vomiting, and intermittent fever unresponsive to antipyretics. She was initially treated for gastroenteritis elsewhere, but persistent symptoms prompted further evaluation. Pelvic CT revealed bilateral TOAs (Figure 1), and she was transferred to our institution for management.

**Figure 1:**
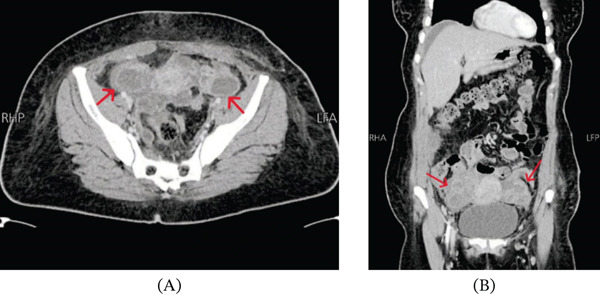
(A) Axial and (B) coronal contrast‐enhanced CT images demonstrate bilateral adnexal abscesses (red arrows) consistent with tubo‐ovarian abscesses.

She had prior episodes of vaginitis and UTIs. Her medical history was significant for poorly controlled DM II (HbA1c 9.7%). The patient′s BMI was approximately 31. Upon admission, the patient complained of severe abdominal pain. After initial laboratory investigations (within 30 min of her presentation), she was found to have diabetic ketoacidosis (DKA) (hyperglycemia [350 mg/dL], positive ketonuria, high anion gap metabolic acidosis, and CRP of 377 mg/L, and WBC count of 9.53). On examination, the patient was ill but hemodynamically stable, with a temperature of 36.5°C. The abdomen was soft and lax with bilateral upper quadrant tenderness. No peritoneal signs were noted. Pelvic ultrasound demonstrated bilateral complex adnexal masses (≈4.4 cm) with internal echoes consistent with TOAs. Ultrasound confirmed the TOAs previously seen on CT. She was admitted to the ICU for intravenous fluids, insulin infusion, electrolyte correction, and broad‐spectrum antibiotics. After initial stabilization, persistent fever and elevated CRP warranted laparoscopic drainage on Day 4. Both ovaries were enlarged and adherent to the tubes, containing purulent material. The abscess cavities were carefully opened using blunt and sharp dissection, followed by suction of the purulent material. The cavities were thoroughly irrigated with warm saline, loculations were broken down, and samples were sent for histopathology and culture. A pelvic drain was placed through the lateral port site. Histopathology confirmed inflammatory abscesses with necrosis. No ovaries or tubes were removed; the procedure focused on drainage. Her antibiotic inpatient course involved vancomycin and meropenem while in the ICU. After stabilization, the antibiotic regimen was tailored to doxycycline, metronidazole, and ceftriaxone based on clinical response. Wound cultures were negative, likely due to prior antibiotic administration, and no predominant organism was grown; however, TOAs typically involve mixed organisms.

Postoperatively, the patient improved markedly with fever resolution, declining CRP (to 90 mg/L), and stable glycemic control. She was discharged in good condition on tailored antibiotics and diabetes management, with follow‐up arranged with endocrinology and gynecology.

## 3. Discussion and Conclusion

Common precipitants of DKA include infections, interruption of insulin therapy, acute cardiovascular events, acute pancreatitis, and certain medications [4]. It is important to emphasize that genitourinary (GU) and gynecologic infections, such as a urinary tract infection (UTI), PID, or a TOA, can also act as credible triggers for DKA. In one epidemiologic series, the most common infection sources precipitating DKA included UTIs and respiratory tract infections [5].

DKA can present with symptoms that closely mimic those of an acute abdomen, including diffuse abdominal pain, vomiting, and fever, all of which are also common in conditions like PID or TOA. The overlap between DKA and pelvic infections can contribute to a delayed diagnosis of the actual source of the abdominal pain, which can result in potentially serious complications if not managed promptly [6, 7].

The coexistence of severe infection, metabolic decompensation, and bilateral TOAs highlights the bidirectional relationship between infection and hyperglycemia: Infection can trigger DKA through counterregulatory hormones and proinflammatory cytokines that increase insulin resistance and ketoacidosis, whereas DKA impairs immune function and delays infection resolution [8, 9].

Several publications have identified key predictors of failure of conservative management in patients with a TOA. A prospective cohort found that a CRP cut‐off of > 49.3 mg/L had 85% sensitivity and 93.4% specificity for predicting TOA (i.e., more complicated PID) and that rising CRP trends early in hospitalization portended the need for invasive intervention [10, 11]. Other documented risk factors include bilaterality of the abscess, persistent fever after 48–72 h of therapy, older age (often > 35 years), and large abscess in several series [12]. When multiple of these features coexist, the likelihood of requiring a drainage procedure or surgical intervention rises substantially [13].

The significantly elevated CRP (377 mg/L) indicates a severe systemic inflammatory response, suggesting that medical therapy alone would be insufficient. This justified the decision to proceed with surgical intervention.

Although the abscess size in this patient (~4.4 cm) might be considered moderate, the bilateral involvement significantly increases the likelihood of treatment failure. When combined with elevated CRP and diabetes, the risk of failed conservative management becomes high. Evidence suggests that even moderately sized abscesses with these risk factors may require early drainage rather than delaying intervention [14].

Interventional radiology (IR) drainage is highly successful for simple, unilateral TOAs, but its effectiveness decreases in complex, multiseptated, or bilateral abscesses because not all locules can be reached or adequately drained. In such situations, laparoscopic surgical drainage is preferred, as it allows precise drainage. Given the complexity of this bilateral TOA and the resources at our institution, laparoscopy was the most appropriate choice and is supported by the literature when IR is unlikely to fully resolve the abscess [15].

Tumor markers, including CA‐125, were not measured in this patient. Although CA‐125 is often used in evaluating adnexal masses, elevated levels are nonspecific and may occur in inflammatory pelvic diseases such as TOA [16] [17].

Before surgery, the patient required correction of acidosis and glycemic stabilization. Achieving euglycemia preoperatively improves postoperative outcomes and decreases the risk of surgical site and other infections. A retrospective cohort study found that patients with preoperative HbA_1_c < 7*%* had significantly fewer postoperative infectious complications [18].

The patient′s uncontrolled DM II predisposes to impaired immune function, increasing infection severity and recurrence risk [19]. In the setting of a bilateral TOA, this risk is compounded, and the absence of bilateral salpingo‐ovarectomy places her at higher risk of recurrence despite initial drainage. Published recurrence rates for TOA range approximately 10%–20%, and diabetes is a risk factor [20]. Given these factors, vigilant long‐term follow‐up and a low threshold for reintervention are advised.

This case emphasizes the importance of early recognition and diagnosis of a gynecologic source of infection as a precipitating factor for metabolic decompensation in diabetic patients, which can dramatically improve outcomes.

## Author Contributions

A.N., H.O.A., B.J.A., H.J.O., and R.S.: writing. H.O.A., B.J.A., and H.J.O.: data collection. H.O.A. and A.N.: literature review and discussion. R.S.: guided the manuscript preparation. M.S. supervised the clinical management.

## Funding

No funding was received for this manuscript.

## Disclosure

All authors read and approved the final manuscript.

## Ethics Statement

The authors have nothing to report.

## Consent

Written informed consent was obtained from the participant included in the study. Consent was also obtained for the publication of anonymized data and any accompanying images.

## Conflicts of Interest

The authors declare no conflicts of interest.

## Data Availability

The data that support the findings of this study are available from the corresponding author upon reasonable request.
